# Efficient CRISPR/Cas9 mediated Pooled-sgRNAs assembly accelerates targeting multiple genes related to male sterility in cotton

**DOI:** 10.1186/s13007-021-00712-x

**Published:** 2021-02-08

**Authors:** Mohamed Ramadan, Muna Alariqi, Yizan Ma, Yanlong Li, Zhenping Liu, Rui Zhang, Shuangxia Jin, Ling Min, Xianlong Zhang

**Affiliations:** 1 National Key Laboratory of Crop Genetic Improvement, Huazhong Agricultural University, Wuhan, 430070 Hubei China; 2 Department of Plant Genetic Resources, Division of Ecology and Dry Land Agriculture, Desert Research Center, Cairo, Egypt

**Keywords:** Cotton, CRISPR/Cas9, Genome editing, Male sterility, Pooled sgRNAs assembly

## Abstract

**Background:**

Upland cotton (*Gossypium hirsutum*), harboring a complex allotetraploid genome, consists of A and D sub-genomes. Every gene has multiple copies with high sequence similarity that makes genetic, genomic and functional analyses extremely challenging. The recent accessibility of CRISPR/Cas9 tool provides the ability to modify targeted locus efficiently in various complicated plant genomes. However, current cotton transformation method targeting one gene requires a complicated, long and laborious regeneration process. Hence, optimizing strategy that targeting multiple genes is of great value in cotton functional genomics and genetic engineering.

**Results:**

To target multiple genes in a single experiment, 112 plant development-related genes were knocked out via optimized CRISPR/Cas9 system. We optimized the key steps of pooled sgRNAs assembly method by which 116 sgRNAs pooled together into 4 groups (each group consisted of 29 sgRNAs). Each group of sgRNAs was compiled in one PCR reaction which subsequently went through one round of vector construction, transformation, sgRNAs identification and also one round of genetic transformation. Through the genetic transformation mediated *Agrobacterium*, we successfully generated more than 800 plants. For mutants identification, Next Generation Sequencing technology has been used and results showed that all generated plants were positive and all targeted genes were covered. Interestingly, among all the transgenic plants, 85% harbored a single sgRNA insertion, 9% two insertions, 3% three different sgRNAs insertions, 2.5% mutated sgRNAs. These plants with different targeted sgRNAs exhibited numerous combinations of phenotypes in plant flowering tissues.

**Conclusion:**

All targeted genes were successfully edited with high specificity. Our pooled sgRNAs assembly offers a simple, fast and efficient method/strategy to target multiple genes in one time and surely accelerated the study of genes function in cotton.

## Background

Cotton is one of the world’s most important economic crops and its importance is mainly based on fiber and oil production [1]. Most of the essential agronomic traits in crop plants like yield are quantitative traits which are controlled by multiple genes and genomic loci [2]. These traits are highly affected by major environmental stresses that limit crop growth and production. Cotton is a highly responsive crop plant to the environmental stresses by which its endurance depends on plant species, genotype and development stage [3]. The development of the reproductive tissues is the most sensitive stage not only in cotton plant but also in most plant species in which any disruption at this stage can cause male sterility [4, 5]. Male sterility is the main challenge that decreases cotton fiber yield [6]. On the other hand, male sterility can be used as a simple pollination control system that is broadly established in cotton hybrid breeding [7]. In recent decades, accumulating transcriptomic and proteomic studies of plant species have identified several genes that play roles in the development of male reproductive organs [8–10]. However, there is still lack of information about the molecular machinery of genes regulating male sterility in cotton. We crucially eager to deeply understand how male sterility occurs and how the regulatory mechanism functions during anther development.

*Gossypium hirsutum* is a polyploidy specie with large genome size (approximately 2.5 Gb) in which most genes have multiple copies and high sequence similarity through At and Dt sub-genomes [11, 12]. This makes cotton genetic engineering and breeding programs quite difficult. Current cotton transformation method targeting one gene requires a complicated, long and laborious regeneration process that goes through 8–12 months of tissue sub-culturing [13]. Therefore, targeting multiple genes is of great value in functional genomics and genetic engineering especially in cotton. In polyploidy species, classical genetic engineering methods such as physical and chemical mutagenesis agents can’t target specific gene and characterize the resulting phenotype which limit its practical application in functional genomic research. Whereas, *Agrobacterium*-mediated T-DNA insertion in such complex genome doesn’t offer the ability to connect phenotype to the genotype due to its low mutation ratio and the random insertion along the genome sequence [14]. The recent accessibility of genome editing tools offers the ability to modify any targeted locus efficiently and provides easy, precious and affordable methods to study the function of many genes in different genomes.

In the last decade, many genome editing tools such as Zinc finger nuclease (ZFNs) ZFNs, Transcription activator-like effector nuclease (TALENs)TALENs, and Clustered Regularly Interspaced Short Palindromic Repeat (CRISPR) have been established [15–19], however; ZFNs and TALENs have limited application in the biotechnology society due to their designing difficulty and assembly [15, 16]. CRISPR/Cas9 system is a new gene editing tool derived from *Bacteria* and *Archaea* immune systems against exogenous nucleic acids, plasmids and phages that can target any DNA sequence. This system contains a powerful, precious and effective nuclease (Cas9) that is activated by a guided-RNA to induce different types of targeted mutations; deletion, insertion, substitutions; resulting in disabling gene function. Owing to its simplicity and high efficiency, CRISPR/Cas9 technology is harnessed to make precise changes in almost whole living specie’s genome like human cells, fruit flies, fish, mice, and plants [22–27].

Next-generation sequencing (NGS) is a main strategy adopted by scientists to create millions of sequencing reactions [28], and generate a valuable amount of sequence information [29]. Although many tools have been developed to analyze the sequencing data [30–33], Hi-TOM is a simple and cheap strategy to identify hundreds of mutagenesis induced by CRISPR/Cas9 technology [34]. In cotton plant, CRISPR/Cas9 has been established with high editing efficiency rate of 87% [2] and low off-target mutations [35]. Although CRISPR/Cas9 in cotton is an effective strategy to study individual targeted genes, there still a few studies have been stated.

The generation of a bulk of targeted mutations would provide validated and sufficient mutants to study the function of large number of genes. It also would ease the way for cotton breeders to better understand and characterize cotton genome. Recent studies in other crops such as rice, soybean, maize, and tomato have established pooled CRISPR/Cas9 methods to generate a population of mutants [36–39].

This notion inspired us to build a CRISPR/Cas9 system mediated pooled sgRNAs assembly targeting 112 genes belonged to different families to find the key genes that may improve fertility in cotton. The assembly was constructed by pooling a mixture of sgRNAs that were designed to target single or duplicated genes in one round of PCR amplification, one round of vector construction and one round of genetic transformation. Our sgRNAs assembly provides a bulk of purposeful mutants that would accelerate our understanding of male sterility in cotton and ensure speedy characterization of the studied genes for cotton genetic improvement in the future.

## Methods

### sgRNAs design

To identify the function of a group of genes in cotton, a pooled sgRNAs assembly using CRISPR/Cas9 system was performed. First, CRISPR-P2.0 web site (http://crispr.hzau.edu.cn/CRISPR2/) was used to determine the sgRNA/s for each gene using *Gossypium hirsutum* TM-1 sequence as a reference genome [11]. Genome-wide comparison screening was performed and total of 116 sgRNAs were selected to target candidate genes (Additional file 2: Table S2). These sgRNAs are usually 23 bp in length considering the following criteria: (i) sgRNA sequences are localized in exon regions of the targeted genes; (ii) the content of GC ranges between 40 and 60%; (iii) sgRNAs target the region near the 5′ end of the genes; (iv) single gene-specific sgRNAs contain at least one mismatch at the 3′ end to avoid potential off-targets. The mismatch value of the target sequence by genome-wide alignment was greater than 2 mismatches.

### Vector construction and induced bacterial transformation

The CRISPR/Cas9 vector used in this study is derived from previous report [2]. Every selected sgRNA was synthesized as a DNA oligonucleotide fused to 16 bp sequences homolog to the linear ends of *pRGEB32- GhU6.9* vector as following: to construct our pooled sgRNA assemblies, plasmid was digested using *BSAI* restriction enzyme for 5 h at 37 °C, 29 of oligo sgRNAs were separately pooled in equal amount then amplified by PCR (primers sequence are shown in Additional file 3: Table S3). The purified PCR products were fused to the linearized *pRGEB32- GhU6.9* vector using ClonExpress II One Step Cloning Kit. Each assembly group should contain at least 200 monocolonies of Bacterial-host-competent cells grown in a selection medium. All resistant cells were then harvested in 100 mL lysogeny broth (LB) medium and cultured over night for plasmid extraction. Next, after plasmids extraction, the yielded plasmids were introduced to *Agrobacterium* (*GV3101*) in which each group contained at least more than 500 monocolonies. Third, for each sgRNA assembly, all grown colonies were collected in 100 mL LB with kanamycin which used for high throughput sequencing to ensure that all targeted sgRNAs are included in the pooled assembly.

### Plasmid extraction and NGS sequencing in *Agrobacterium*

To make sure that all sgRNAs were covered, the plasmid DNA of the pooled sgRNAs library was extracted from the agrobacterium according to TIANprep Rapid Mini Plasmid Kit (TIANGEN, cat. no. 4992191/4992192), PCR reactions were performed using specific primers to amplify the sgRNAs that integrated into this vector, and the PCR products were sequenced (Primers sequences are shown in Additional file 3: Table S3).

### Plant material for *Agrobacterium*-mediated transformation in cotton and growth conditions of the regenerated plants

For each pooled sgRNAs assembly, transgenic cotton plants were generated by the *Agrobacterium tumefaciens*-mediated transformation. 100 seeds from an elite cotton (*Gossypium hirsutum*) cultivar Jin668 grown in dark were treated according to a conventional protocol [35]. The cotton plants, *G. hirsutum* L. cv. Jin668 and transgenic lines of CRISPR/Cas9 -mutated plants were planted in the greenhouse (20–25 °C in the night and 28–35 °C in the day time, under a 16/8 h light/dark photoperiod) in commercially sterilized soil (a complex of soil, peat, and composted pine bark).

### Barcodes design for genomic DNA sequencing to identify sgRNAs in the mutants

Genomic DNA of the total transgenic plants was extracted. Primers were designed including sgRNA region from plasmid sequences and DNA barcodes with six nucleotides were added to 5′ end of primers. A total of 12 forward and 32 reverse primer’s barcodes were designed to detect 384 samples. Then, the 300-bp PCR products were generated from all plant’s DNA. Final reaction products were analyzed with 1% Agarose gel electrophoresis. All the products were purified and mixed with equal Nano mole as one sample for DNA library construction using Illumina Truseq DNA sample preparation kit (Illumina, San Diego, CA) according to manufacturer’s instruction. It is applied to the Illumina HiSeq 3000 system (paired-end 150 bp reads, Illumina).

### Hi-tom and gene editing efficiency

To detect editing efficiency, PCR was performed to amplify the targeted genomic DNA by a pair of site-specific primers with common bridging sequences (5′-ggagtgagtacggtgtgc-3′ and 5′-gagttggatgctggatgg-3′) added at the 5′ end. The primary amplification of 96 samples was performed in a 20 µl reaction volume containing 1 µl of genomic DNA, 0.3 µM of specific forward and reverse primers (1 µm), 2 µl easy Tag buffer, 0.2 µl Tag polymerase, 0.4 µl 10 mM dNTPs and up to 20 µl ddH_2_O. The secondary amplification was conducted in 20 µl preassembled kits, each containing 10 µl 2 × Taq Master Mix, 200 nM 2P-F and 2P-R primers, 2 nM F-(N) and R-(N) primers (Additional file 3: Table S3), and 1 µl primary PCR product. PCR conditions were 5 min at 94 °C (1 ×), 30 s at 94 °C, 30 s at annealing temperature and 25 s at 72 °C (32 ×), followed by 72 °C for 5 min. All the products were purified and mixed with equal Nano mole as one sample for DNA library construction. Hi-TOM web site was used to analyze the data sample-by-sample and export the results in Excel format.

## Results

### Selection of single guided-RNAs (sgRNAs) to target genes related to anther response towards high temperature stress

A total of 112 genes were selected from our reported transcriptome data [40] that were suggested to play a vital role in the response of male reproductive organs to high temperature stress in cotton. The selected genes and their predicted function according to CottonFGD website (https://cottonfgd.org/) are shown in Additional file 1: Table S1. To better understand the function of these genes, save time and minimize the labor work, we generated a collection of targeted mutants using CRISPR/Cas9 mediated pooled sgRNAs assembly. For pooled sgRNAs assembly construction, 116 highly specific single guide RNAs (sgRNAs) were designed downstream the start codon of candidate genes. These sgRNAs are 23 bp in length ending-up with NGG protospacer adjacent motif sequence. Every selected sgRNA was synthesized as a DNA oligonucleotide fused to 16 bp sequences homolog to the linear ends of *pRGEB32- GhU6.9* vector [2]. Out of the 116 sgRNAs, 13 sgRNAs were designed to specifically match single site sequences; 83 sgRNAs were perfectly matched with only 2 specific sites; 14 sgRNAs targeted precisely 3 sites and finally, 6 sgRNAs were considered to target more than 3 sites along the cotton genome. In this study, almost all genes were disrupted by at least more than one sgRNA targeting different coding regions (sgRNA sequences are attached in Additional file 2: Table S2).

### Optimal construction of pooled sgRNAs assemblies led to high coverage of sgRNAs

**Fig. 1:**
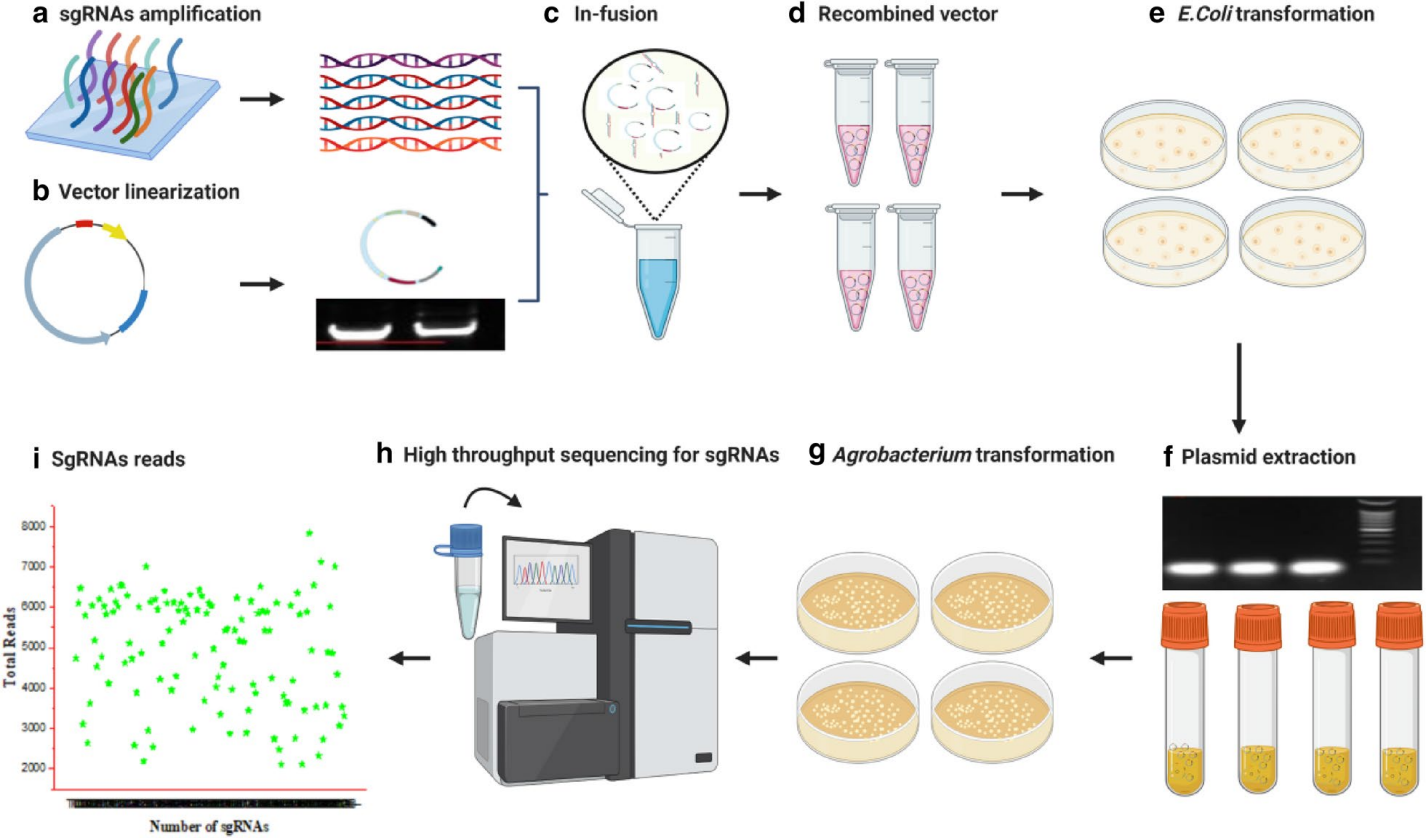
Strategies of constructing the pooled sgRNAs assemblies for cotton genetic transformation. **a** Amplifying the 29 oligo sgRNAs by PCR and PCR products obtained double-stranded sgRNAs. **b** Circular vector and linearized vector. The vector was linearized by *Bsa*I restriction enzyme, the removed part is 300 bp in size. The cut run faster than the control sample (CK) then the linearized sample was purified and used for ligation step. **c** In-fusion reaction contains double-stranded sgRNAs and linearized plasmid. **d** The recombined vector harboring the double-stranded sgRNAs. **e** Clones resulted from the transformation to *E.coli*. **f** Extraction of plasmid from Kanamycin-resistant clones. The size of the resulted positive clones is less than 100 bp. **g** Clones resulted from the transformation to *Agrobacterium*. **h** High throughput sequencing for the pooled assembly of the constructed vectors harboring the targeted sgRNAs. **i ** Large scale sequences in *Agrobacterium* and coverage of sgRNAs. Each star indicates reads of every sgRNA included in this study (This figure was created by BioRender web tool)

Vector construction for every individual gene is costly and time-consuming. To improve vector construction efficiency, the 116 designed sgRNAs were divided into 4 separate assembled groups (29 sgRNAs for each group); each group of the oligo sgRNAs was separately pooled in equal amount and amplified by PCR (Fig. 1a); the PCR product and the linearized *pRGEB32- GhU6.9* vector (Fig. 1b) were next mixed together and fused to form the recombined vector (Fig. 1c and d). Resulted from In-fusion, the fused constructs were introduced to bacterial-host-competent cells (*Top10*) and at least 200 of the positive monocolonies were harvested (Fig. 1e and f). After that, plasmids of the bacterial cells harboring the constructed vector were introduced into *Agrobacterium* (*GV3101*) strain (Fig. 1g). Overall, each assembled group contained not less than 500 *Agrobacterium* monocolonies. Finally, all grown colonies were collected and used for high throughput sequencing to ensure all targeted sgRNAs are included in the pooled assembly (Fig. 1h).

### Large scale sequences in *Agrobacterium* to check the coverage of sgRNAs during pooled construction

Coverage and uniformity in vector construction are the main factors for successful genetic transformation during *Agrobacterium-*mediated plant transformation. Therefore, all grown colonies presented in the cultured Petri plates were collected for plasmid extraction. Because almost all constructed plasmid sequences were the same except those of the spacer sequences (23 bp) that showed the differences between each vector, primers from flanking sequences were used to amplify all these sequences. A 150 bp upstream and downstream the sgRNAs inserted in *pRGEB32- GhU6.9* vector of the four assembled groups were amplified and collected together in one tube for next-generation sequencing (NGS). Sequencing results showed that all the constructed 116 sgRNAs were existed; the coverage of sgRNAs was successfully 100%. The sequencing reads were obtained between 2000 and 7000 for each sgRNA (Fig. 1i). This variation in the sgRNAs reads might be due to many factors such as PCR conditions, priming and buffers quality of the purification Kit or sequencer. However, most of the reads ranged between 4800 and 5300. These result indicated that pooling 29 sgRNAs in one assembly is suitable for a successful, faster, easier and lower cost than single vector construction.

### *Agrobacterium* transformation in cotton using constructed pooled sgRNAs assemblies

**Fig. 2:**
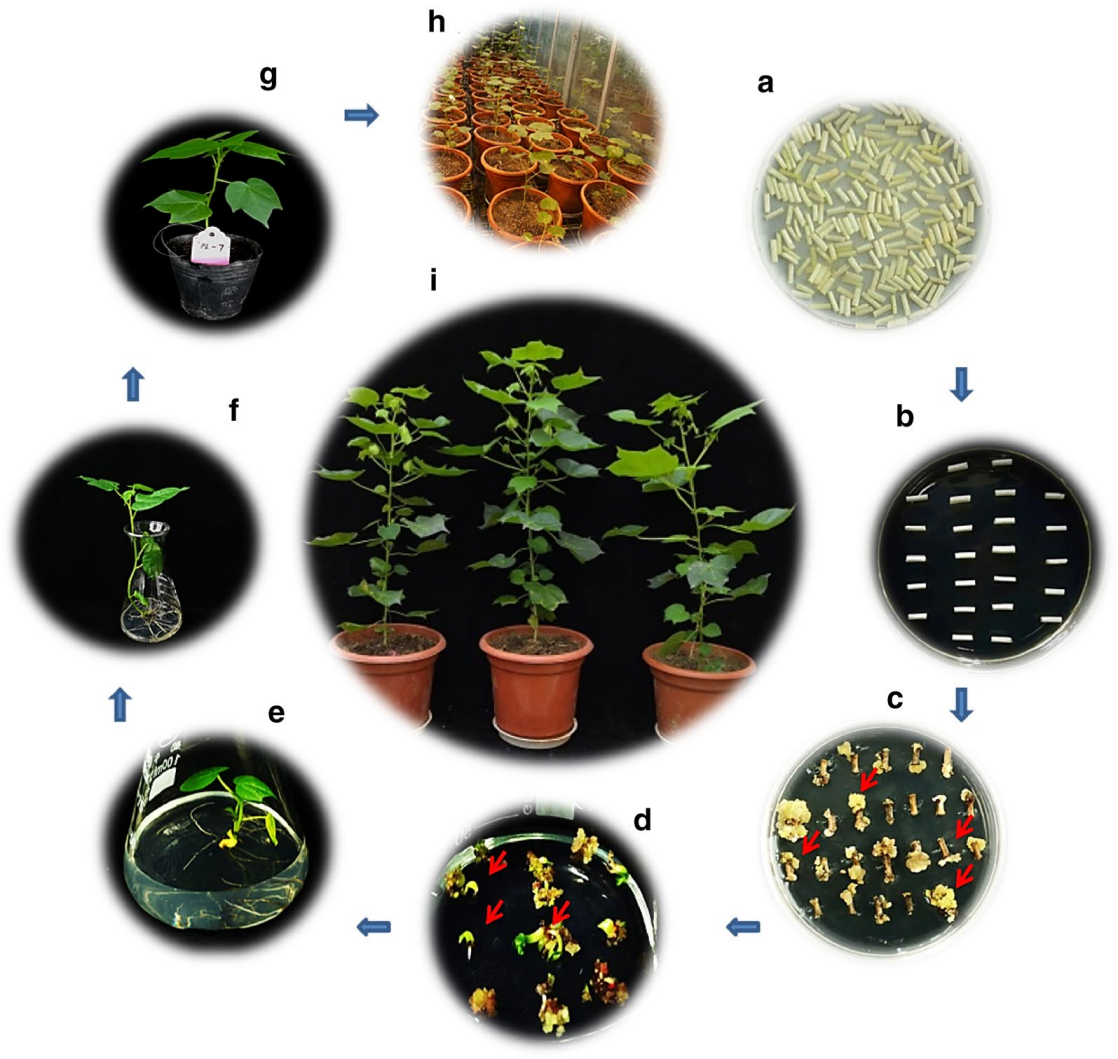
Different stages of tissue culture during the generation of CRISPR-Cas9 pooled sgRNA assembly mutants by *Agrobacterium*-mediated transformation in cotton. **a** Transformed hypocotyls were placed on co-culture medium after treated with Agrobacterium suspension. **b** Transformed hypocotyls on 2,4 D medium containing kanamycin and cephalosporin antibiotics. **c** Hypocotyls after 4 months of transformation on 2,4D medium. **d** Two months old differentiated calli on differentiation medium. **e** Plantlets were transferred into rooting medium. **f** Transgenic plants into the Hoagland medium for more adaptation. **g** Transgenic plants were transferred into small pots. **h**, **i** Transgenic plants were grown into big pots in the greenhouse

Independently, the genetic transformation of cotton has been done for each pooled sgRNAs assembly using 150 seedlings of Jin668 cultivar as explants. Transformants went through a series of subculture processes starting with the co-culture at 20 °C for 48 h in the dark (Fig. 2a) then shifted to medium containing 2,4-D for callus induction (Fig. 2b). At least 2000 hypocotyl segments (length ≤ 0.8 cm) were used for callus induction (about 80 Petri dishes). Out of 2000 hypocotyl segments (Fig. 2c), approximately 600 single positive calluses (somatic embryogenesis) were shifted to differentiation medium for cells differentiation (Fig. 2d). After sub-culturing of differentiation, about 200–300 normal plantlets were shifted to the rooting medium (Fig. 2e), then gradually acclimated to the normal conditions (Fig. 2f). In sum, we harvested more than 800 differentiated plantlets from all pooled sgRNAs assembly groups (Fig. 2g). Out of them, 718 T0 plants were successfully shifted to the greenhouse (Fig. 2h and i) for phenotyping and genotyping analyses.

### Barcode-based high-throughput sequencing is a powerful strategy to identify inserted sgRNAs in CRISPR/Cas9 mutants

**Fig. 3:**
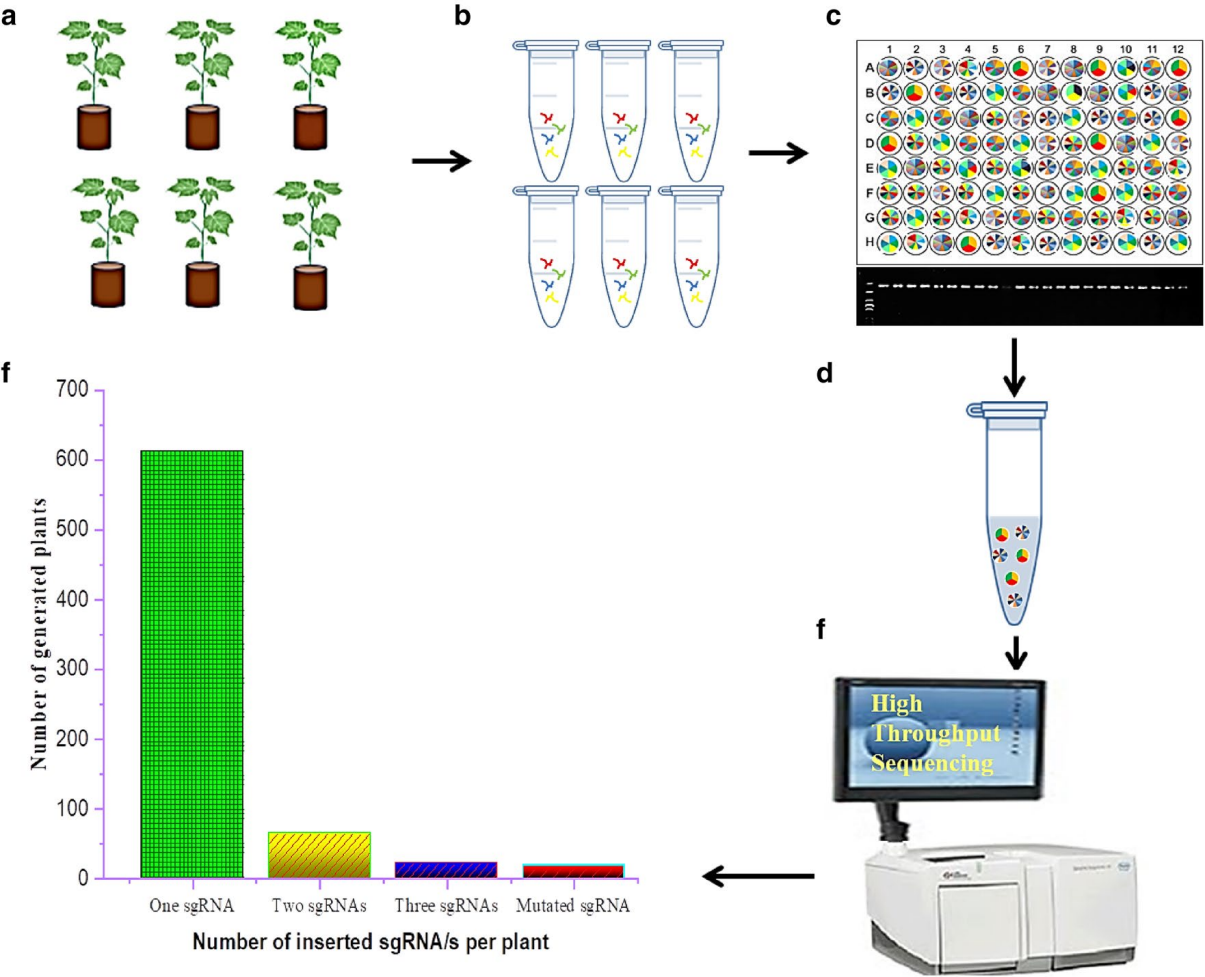
Graphic drawing of the workflow of barcoding.** a** Schematic represent generated plants used for barcoding.** b** The genomic DNA of every generated plants was extracted individually. **c** DNA used for PCR-based barcodes library construction went through one round of amplification and each sample was labeled independently with unique barcodes. **d** DNA library of all tested plants. DNA library was built by pooling the positive PCR mixture together as one sample in equal amount and were adjusted to NGS. Positive PCR results indicated the presence of the T-DNA insertion harboring the sgRNA. **e** High throughput sequencing for the DNA library. **f** Statistics of high throughput sequencing results identified number of sgRNAs existed in each plant

Doubtlessly, PCR-based barcodes library is a viable method that offers high-throughput sequencing of hundreds of gene loci in one pooled sample at once. Barcoding strategy has been used for tracing DNA or RNA that is originated from separate cells or individuals [41, 42]. Our optimized CRISPR/Cas9 system has generated a total of 718 transgenic plants harboring undefined sgRNAs/insertions. To detect and identify the inserted sgRNAs for such number of generated mutants is very challenging. Therefore using barcode sequencing rather than Sanger sequencing is of great significance in which it saves time, labor and money. As a result, we designed different barcodes at 5′ end of the primers, and a total of 44 primers can simultaneously detect the sgRNA sites of 384 mutants. Barcodes and primers sequences are shown in Additional file 3: Table S3. The genomic DNA of all generated plants was used for PCR-based barcodes library construction in one round of amplification (Fig. 3a and b). Each mutant was labeled individually with unique barcodes at both 3' and 5' ends via PCR, positive PCR results indicated the presence of the T-DNA insertion harboring the sgRNA (Fig. 3c). PCR results showed all generated mutants harbored the sgRNA insertion. After PCR confirmation, DNA library was built by pooling the positive PCR mixture together as one sample in equal amount; one sample included 384 individual DNA fragments (Fig. 3d). Subsequently, the pooled DNAs products comprised the assembled sgRNAs were adjusted to Illumina HiSeq 3000 system for paired-end 150 bp reads (Fig. 3e). High throughput sequencing results identified which sgRNA exists in each mutant. The results showed that all the sequenced individuals harbored T-DNA with the targeted corresponding sgRNAs and no mock insertions were observed. Out of 116 designed sgRNAs, 83 sgRNAs were existent in the mutated population, and all the identified sgRNAs covered all the targeted genes. Among 718 T0 plants, 613 harbored only one sgRNA; 65 plants contain two different sgRNAs; 22 plants contain three different sgRNAs and 18 plants contain mutated sgRNA that might be caused during PCR amplification or due to impurity of the synthesized sgRNAs (Fig. 3f). The huge number of plants (85% of total plants) with only one sgRNA indicated that our pooled sgRNAs assembly is efficient enough to induce targeted mutations with high possibility to produce more independent lines in a short time at low cost. As a result, we can infer that CRISPR/Cas9 mediated pooled sgRNAs assembly is a powerful strategy which may pave the way for functional genomic researchers to study multiple genes and their homologues not only in cotton but also for those plant species having complex genomes.

### Tracking mutations by Hi-Tom based NGS sequencing and gene editing efficiency analysis

**Fig. 4:**
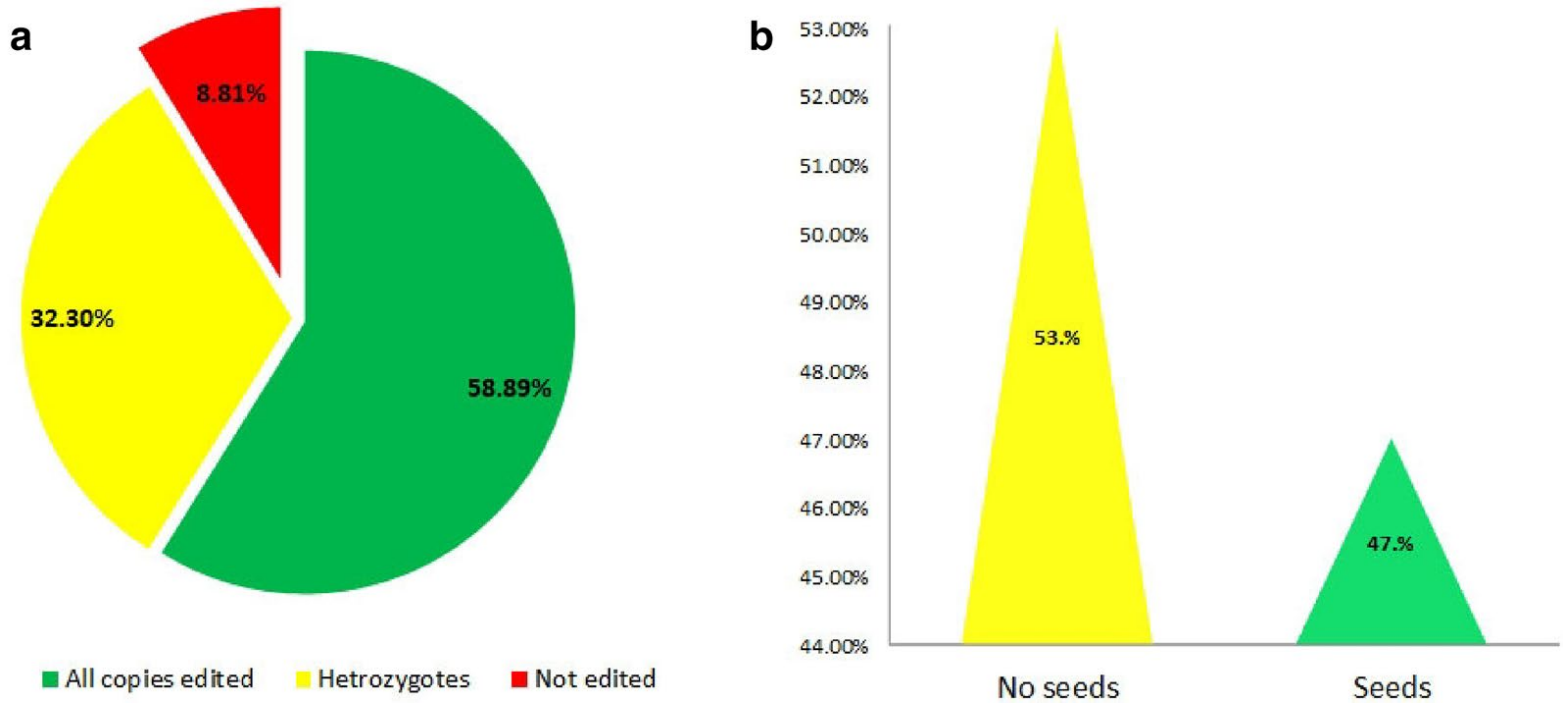
Hi-TOM sequencing results and total seeds production of the plants harboring single sgRNA target. **a** Editing frequency statistics using Hi-TOM strategy. ** b **Total seeds production statistics of CRISPR/cas9 cotton mutants

Although many tools have been developed to track mutation type induced by genome editing tools [31–34, 44]. Hi-TOM is a simple and cheap strategy to detect hundreds of mutants induced by CRISPR/Cas9 technology [34]. After the identification of sgRNA/s located in each transgenic plant, we used high-throughput (Hi-Tom) sequencing method for mutations profiling for each generated plant. In this study, 613 genomic DNA samples were used individually to amplify the targeted region of each candidate gene using site-specific primers. After that, the targeted regions went through second round of amplification to label each plant by unique barcodes. The final size of the DNA fragment resulted from the two rounds of PCR was about ~ 200 bp. Every 96 PCR individuals were pooled together as one sample for sequencing and analysis. Sequencing results showed that all candidate genes were successfully edited; 361 plants exhibited editing at the directed sites driven by our sgRNAs that formed (58.89%) over all plants (Fig. 4a). The editing achieved various targeted mutagenesis of our candidate genes in which deletions accounted the highest proportion. Heterozygous mutations also took a part in our population, there are 198 plants displayed editing whether in only one allele or in double alleles at different sites. Only 54 plants showed no editing in their targeted genes. The results indicated that our CRISPR/Cas9 mediated pooled sgRNAs assembly is a highly effective strategy to preciously edit wide number of genes in one round of transformation.

### Mutation characteristics of the generated plants

Our pooled sgRNAs assembly included genes proposed from transcriptome data in the regulatory of the male reproductive organ development in cotton. By our CRISPR/Cas9 mediated pooled sgRNAs assembly, these genes were successfully knocked out and a huge number of the mutants were generated. Therefore, once the mutant is obtained, it is necessary to screen all the generated lines harboring different genes for any changes in their growth or productivity compared to non-transgenic plants.

### Aberrant morphological phenotypes

**Fig. 5:**
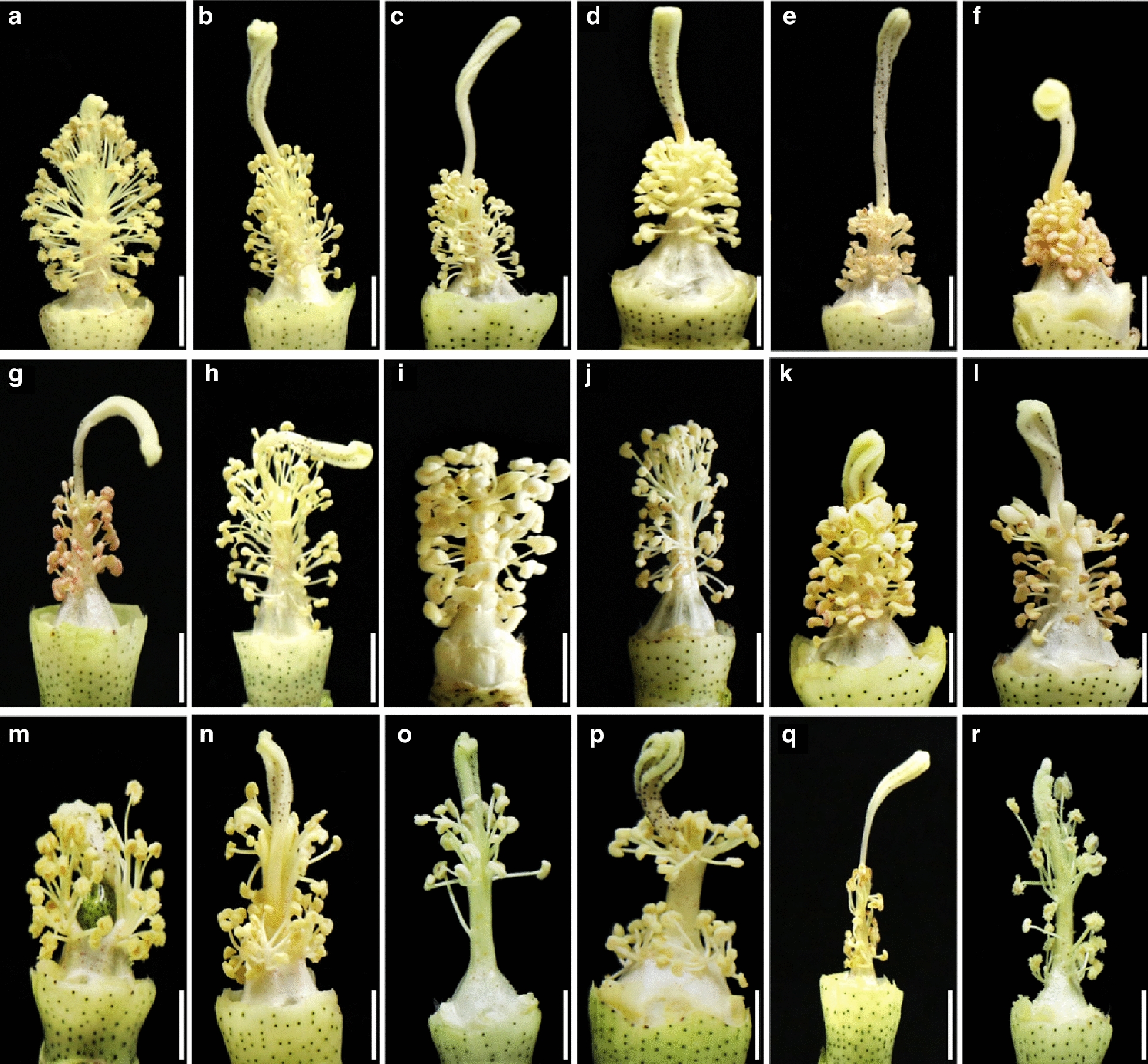
Comparison of the anther structure in JIN668 (CK) and different mutant lines generated by the sgRNAs pooled assembly.** a** Represent normal anther structure of the JIN668 (CK) as a control.** b**–**h** Represent long-sterile anther phenotype in different mutant lines. **e**–**g** Represent shrivel anthers in different mutant lines. **i**, **j** Represent short stigma and non-dehiscent anthers in different mutant lines. **k**–**n** Represent abnormal style phenotype in different mutant lines. **o**–**r** Represent few filaments phenotype in different mutant lines. White lines indicates the scale bar = 1 cm (**a**–**r**)

**Fig. 6:**
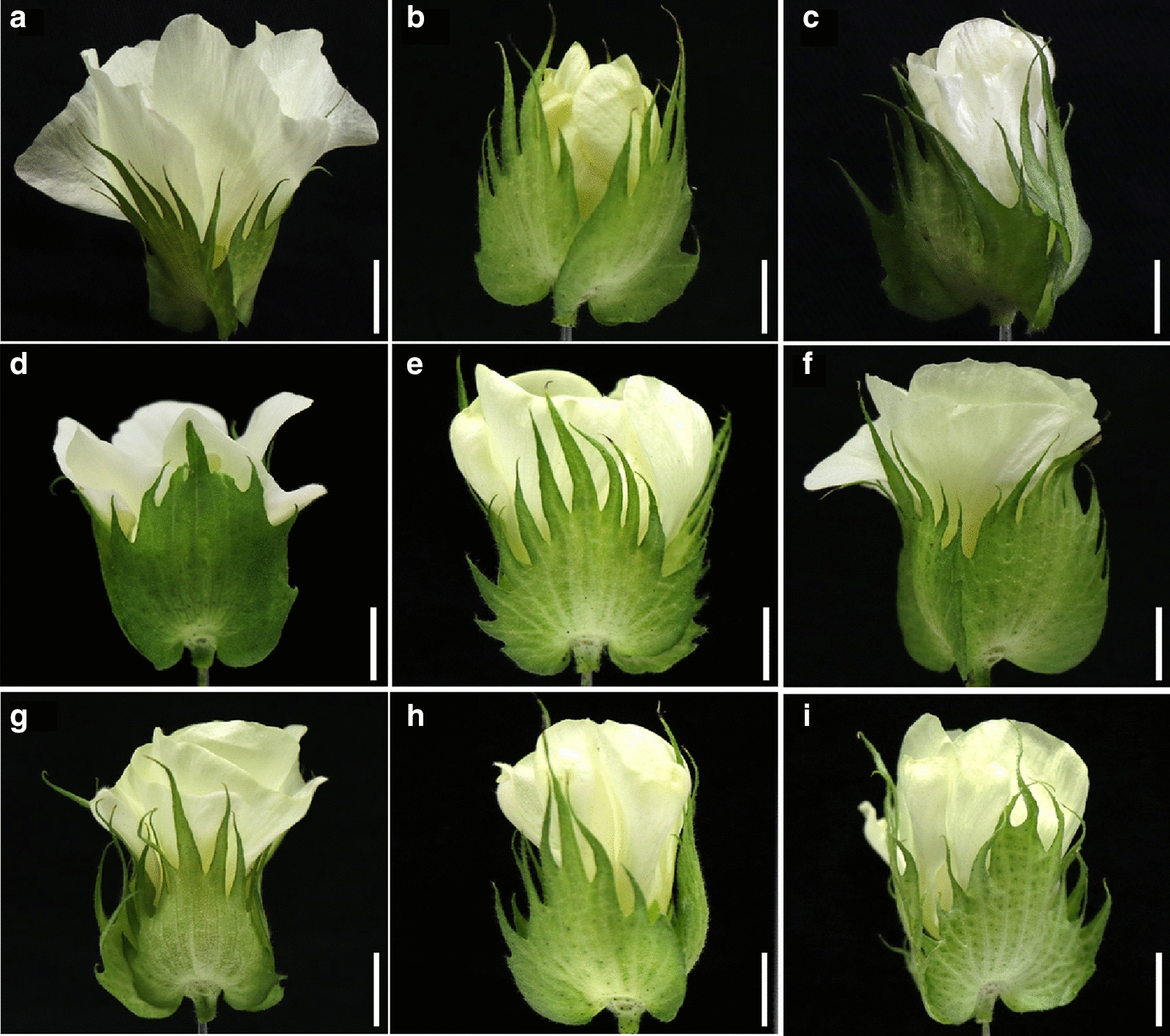
Comparison of the flowers in JIN668 (CK) and different mutant lines generated by the sgRNAs pooled assembly. **a **Represent normal flower shape of the JIN668 (CK) as a control. **b**–**d** Represent flowers with small petals phenotype in different mutant lines. **e**–**i** Represent small flowers phenotype in different mutant lines. White lines indicates the scale bars =1 cm (**a**–**l**)

All transgenic plants harboring single targeted sgRNA were compared with Jin668 (as control) in the greenhouse under normal growth conditions. Phenotyping was conducted by the screening of 613 independent lines containing 112 genes that were successfully edited. Through morphological comparison between the wild-type and library mutants, several floral phenotypes were observed among the progeny of the transgenic plants (Fig. 5a), such as long stigma (Fig. 5b–h), short stigma and non-dehiscent anthers (Fig. 5i–m), shrivel anthers (Fig. 5e–g), fewer filaments (Fig. 5o–r), small flower and small petals (Fig. 6). These phenotypes might be a key to understand the molecular mechanisms of anther and pollen development in cotton, which there still few studies focused on this research area. These results also indicated that this system can generate wide scale of genotypic and phenotypic mutagenesis.

### Seeds production ability in the generated plants

One of the main targets of culturing T0 plants is to collect seeds for the next generation to study T1 progeny. For that, the generated plants were shifted to the greenhouse and seeds production has been screened/noted in all studied lines for two times (after 3 months and 6 months of shifting time). Interestingly, more than 53% of the population of mutated plants couldn’t produce seeds (Fig. 4b). Notably, plant failed to produce seeds obtained homozygous mutants. The failure of seeds production most probably might be due to mutation induction or less likely because of the somaclonal variations. These results demonstrated that our library may provide a good resource for further deep functional genomic studies especially in the field of reproductive traits in cotton. Also it opens new insights for functional genomic research area to study multiple genes in a short time.

## Discussions

### Efficient CRISPR/Cas9 mediated pooled sgRNAs assembly is applicable tools to study complex genomes and obtain a numerous of mutants

In addition to many major crops and horticultural species, CRISPR/Cas9 system has been successfully established in cotton plant exhibiting high editing efficiency and low off-target mutations [2, 44]. At the same time, RNA seq and proteomic data offer and propose a bulk of genes related to different traits. However, there is a huge gap between the advances achieved by the molecular biology research and the number of studies reported in the cotton functional genomic area. This gap refers to the complexity to produce transgenic cotton plants, studying a single gene is a time-consuming process with fewer outcomes. Hence, the need of a strategy that accelerates specific gene targeting and generates purposeful mutations would be a great value in the functional genomic research area especially in those crops with complex genomes such as cotton.

Recently, mutagenesis targeting many genes along the genome or a gene family were demonstrated in major crops such as tomato, rice, soybean and maize using CRISPR system [37, 39, 45, 46]. However, CRISPR/Cas9 mediated large-scale mutation focusing on a certain trait has not been reported in plants yet. This idea inspired us to use the advantage of CRISPR/Cas9 mediated large-scale mutation to focus on one of the main problems in cotton plant production which is male sterility.

Our strategy focused on decreasing the cost, labor and time that makes it useful not only to screen multi-genes but also to discover elite genes by designing highly specific sgRNAs that target specific genes and their duplications. Unlike single sgRNA construction of soybean [37], we compiled 29 sgRNAs together as one sample which went through the same steps of single sgRNA construction that allowed us to all targeted genes were covered in our pooled assembly with less time and labor.

Additionally, one of the main advantages of multiple gene library construction is to generate a large number of plants. However, an excessive number of generated plants would be useless due to the difficulty to manage and evaluate. In our mini-library, we generated 713 plants that covered all the 112 targeted genes. This number of plants is reasonable in which achieved the coverage of all targeted genes and eased plant management and analysis. Comparing our results with the rice report that obtained a huge number of plants, in which the library generated about 14,000 transgenic plants, out of which only 0.22% of plants have been analyzed [39].

Barcoding and Hi-Tom mediated NGS are efficacious mutation-tracking strategies that offer a high coverage of hundreds of genomic loci with low cost compared to Sanger sequencing. The barcoding strategy was used for sgRNAs identification in the generated mutants in which 384 mutants were included in one sample. This allowed us to screen the generated mutants using only two samples. In constant, in the soybean library, the identity of sgRNA was identified using Sanger sequencing and only 20 lines have been studied [37].

From barcoding analysis results, more than 85% of the generated mutants harbored only one insertion of targeted sgRNA which is another advantage in our optimized pooled assembly and none of the previous reports recorded such specificity. On the other side, Hi-Tom strategy was used to track the mutation type in those plants with one insertion and remarkably more than 58% of the targeted genes showed editing in all gene copies. Our system has been optimized to reach the maximum advantage of library construction which can use not only in cotton but in all crops with gene redundancy. Overall, the generated mutants with related genes would open new insights to understand the molecular mechanism of male sterility in cotton.

### Pooled sgRNAs assembly is useful to study gene duplication and screen genes participated in cotton male sterility

Although there many genes in cotton genome have been identified to participate in male sterility, the molecular mechanism of the interaction between these genes and their regulatory mechanism is still relatively lacking. Subsequently, our study targeted one trait related to male sterility. It offers wide selection platform by designing different sgRNAs to target different positions, some targeted single gene copy, some targeted two gene copies while others can target more than three gene copies, all together were constructed in one reaction rather than single vector construction. We successfully generated a wide scale of genotypically and phenotypically mutagenesis related to cotton male sterility using CRISPR/Cas9 mediated pooled sgRNAs assembly. After the genotyping analysis, the generated plants were screened for phenotyping under normal conditions and interestingly more than 53% of plants were completely sterile and failed to produce seeds. This is a clear indicator that most of the candidate genes in our pooled assembly play a vital role in the regulation of cotton male sterility.

On the other hand, although approximately 47% of generated plants exhibited fertile phenotype and were able to produce seeds, these plants might show response under extreme conditions like high temperature. They also can be used for further analyses to understand their role in male sterility. Overall, the method of CRISPR/Cas9 mediated pooled sgRNAs assembly with large-scale mutants is a simple/easy way to study gene duplication and determine the governing genes that play key roles in plant development and productivity.

### Problems and future perspectives to improve the efficiency of sgRNAs assembly in cotton

The promising pooled sgRNAs assembly provided a rapid, easy and fast way to target many genes. Its distinct advantages over a single transformation method enabled us to generate lots of mutants in a short time. To reach the maximum advantages of library construction, there are important points should be considered.

Random gene selection is not recommended, it will accelerate the work load by studying non-significant genes. Selecting specific genes related to a certain trait would be more useful. Thus, before selection, it is suggested to use bioinformatics tools and omics data to predict some novel and promising genes.

Designing sgRNA is one of the key steps should be taken into consideration in library construction. For optimal sgRNAs design, GC content, off-target site ratio and number of mismatch sequences should be considered [47–50]. Selection of CRISPR/Cas9 target site has many restrictions like NGG PAM sequence requirements. Thus, using alternative tools, CRISPR/Cas9 paralogs such as Cpf1 and C2c1 would increase the selected sgRNA platform and broaden the functional studies whether of single gene or large scale libraries of gene editing in cotton. On the other hand, to cover all targeted sgRNAs during transformation steps, it is suggested that combine every 20–25 targeted sgRNAs as one pooled sgRNAs assembly. At the bacterial transformation stage, the number of grown colonies should be more than 200 colonies (*E.Coli* strain) and 500 colonies (*Agrobacterium* strains) per plate (two replications for one assembly) and agrobacterium growth rate before transformation leads to decrease/increase of the inserted sgRNA in each mutant. Double and triple insertion of sgRNAs in one plant took apart of our generated plants and sometimes it is useless, so to avoid this case, we can combine the homologous genes in one assembly to increase diversity between these mutants in which would ease studying genes duplication by generating double mutants of deferent genes in the same mutant.

Cotton is one of the most complex plant species and generating transgenic plants is quite challenging. Consequently, for functional studies, we need to generate an excessive number of mutants for each gene. Another problem is that the percentage of homozygous mutations among the generated population is quite low. Therefore, the use of optimal promoters that can enhance the expression of Cas9 would increase Cas9 editing efficiency in cotton. Subsequently, it might increase the ratio of homozygous mutants over heterozygotes and chimeric phenomenon and would reduce the need to generate a large number of transgenic plants.

## Conclusion

In this study, we optimized a CRISPR/Cas9 mediated pooled sgRNAs assembly in cotton. Our study demonstrated that using pooled sgRNAs assembly offers a wide selection platform in the designing of sgRNAs to target specific single and multiple genes. The ability to construct many sgRNAs in one reaction rather than a single vector construction is viable. CRISPR/Cas9 mediated pooled sgRNAs assembly can be adapted not only in cotton but also for all plant species, especially those have complex genomes. The proposed work will not only contribute to the cotton male sterility research area but also provide genetic resources for improving different traits on the field level.

## Supplementary Information

**Additional file 1.** List of the selected genes and their predicted function.

**Additional file 2.** List of the selected sgRNAs and their corresponding genes.

**Additional file 3.** List of primers sequences used in this study.

## Data Availability

The datasets used and/or analyzed during the current study available from the corresponding author on reasonable request.
